# Ethnic disparities in the immune microenvironment of triple negative breast cancer and its role in therapeutic outcomes

**DOI:** 10.1002/cnr2.1779

**Published:** 2023-01-12

**Authors:** Kelsee K. Zajac, Saloni Malla, Ramapuram Jayachandra Babu, Dayanidhi Raman, Amit K. Tiwari

**Affiliations:** ^1^ Department of Pharmacology and Experimental Therapeutics The University of Toledo Toledo Ohio USA; ^2^ Department of Drug Discovery and Development, Harrison School of Pharmacy Auburn University Auburn Alabama USA; ^3^ Department of Cell and Cancer Biology University of Toledo Health Science Campus Toledo Ohio USA

**Keywords:** breast cancer, chemokines, metastasis, racial disparity, therapeutics, TNBC, tumor microenvironment

## Abstract

In 2020, newly diagnosed breast cancer (BC) cases surpassed that of lung cancer among women, making it the most common female cancer globally. In spite of recent increases in incidence rates, mortality due to BC has declined since 1989. These declines have been attributed to advancements in treatment modalities as well as increased mammography surveillance. Despite these advances, African American (AA) women are 40% more likely to die from BC than Caucasian women. Multifactorial etiology has been implicated in the disparity of BC mortality rates among AA women. As an example, AA women have a disproportionate incidence of triple negative breast cancer (TNBC), which has a poor prognosis and marginal treatment options. Increasingly, the tumor microenvironment (TME) has gained relevance as it relates to primary tumor progression, metastasis and treatment possibilities. The treatment outcomes or pathological complete response (pCR) in TNBC among AA women are affected by differences in TME. The TME of AA women exhibit several variances in acellular and cellular components associated with pro‐tumorigenic effects. For example, increased levels of the adipocyte‐related hormone, resistin, the pro‐inflammatory cytokine, IL‐6, and the CC chemokine, CCL2, within the TME of AA women gives rise to an increased density of M2 macrophages, also known as tumor‐associated macrophages. Elevated levels of vascular endothelial growth factor in the TME of AA women increase the vascular density or vascularity, which facilitate aggressive tumor growth and metastasis. Furthermore, a pro‐tumorigenic TME is supported by increased levels of the CXC chemokine, CXCL12 that results in the recruitment of regulatory T lymphocytes (T_regs_). Due to these and other differences in the TME of AA women, precision oncology can target specific aspects of the TME that may contribute to a poorer prognosis. In addition to the discrepancies in the TME, AA women face socio‐economic barriers that limit their ability to access state‐of‐the‐art, novel therapies against metastatic TNBC. In this review, we will provide a brief overview of the tumor immune microenvironment, immune‐based treatment options for TNBC and their potential to decrease health disparities due to ethnicity.

## 1 INTRODUCTION

**FIGURE 1:**
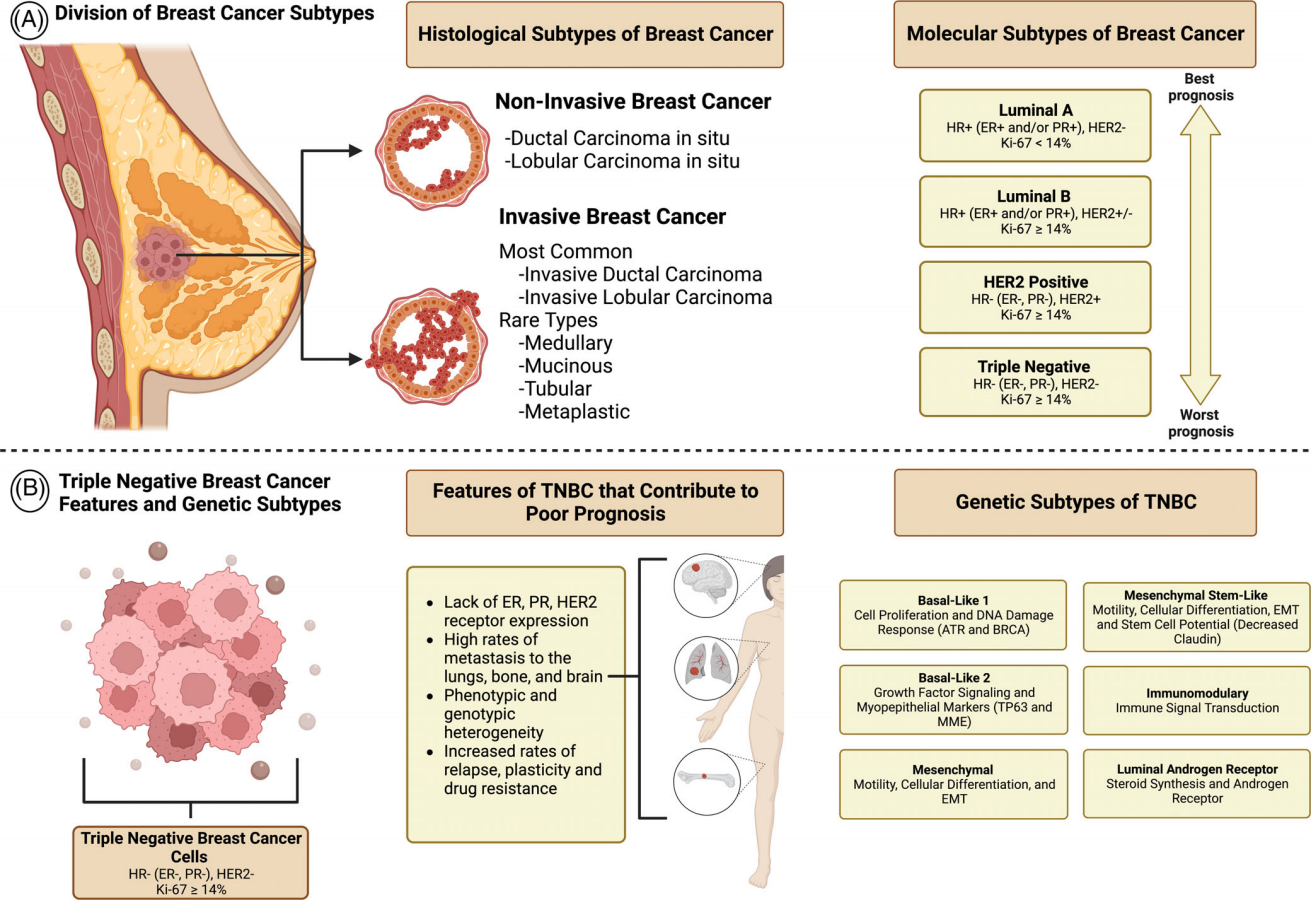
Breast cancer (BC) subtype delineation and a closer look into the triple negative breast cancer (TNBC) subtype. (A) Represents the various histological and molecular delineations of BC subtypes. Invasive BCs may be molecularly grouped into subtypes such as luminal A, luminal B, HER2 positive, and triple negative depending on its receptor and KI‐67 protein expression. (B) This diagram depicts the unique features of TNBC that contribute to its associated poorer prognosis. Based on the gene expression profile, TNBC is further classified into basal‐like 1 (BL1), and 2 (BL2), mesenchymal (M), mesenchymal stem‐like (MSL), immunomodulatory (IM), and luminal androgen receptor (LAR) subtypes

Across the globe, cancer remains one of the leading causes of death among all individuals. In women, breast cancer (BC) is the most diagnosed and greatest cause of female cancer‐related mortality worldwide.^1^ According to estimates for 2022, BC will account for 31% of all new cancer cases, excluding skin, and 15% of those with BC will succumb to their diseases.^2^, ^3^ Mortality in BC patients is primarily due to metastasis to the lungs, bone, and brain.^4^, ^5^ As a heterogeneous disease, BC can be divided into non‐invasive and invasive subtypes. There are two types of invasive BC: invasive ductal carcinoma (IDC) and invasive lobular carcinoma (ILC) which are characterized by the infiltration of cancer cells into normal tissue.^6^ Further classification of invasive BC comes in the form of molecular subtypes based on expression of specific receptors and the Ki‐67 protein. Estrogen receptor (ER), progesterone receptor (PR), and human epidermal growth factor receptor 2 (HER2) expressed alone, in combination, or complete lack of expression make up the invasive subtypes: luminal A, luminal B, HER2+, and basal‐like.^7^ Among the rarer invasive BCs are medullary, tubular, metaplastic, and mucinous carcinomas.^6^ Figure 1A depicts BC histological categories and molecular subtypes, such as invasive molecular subtypes and their receptor and Ki‐67 protein expression variations. Luminal BC is more responsive to targeted hormone therapy such as estrogen modulators (tamoxifen), anti‐HER2 receptor blockers (trastuzumab), and aromatase inhibitors (letrozole and anastrozole).^8^, ^9^, ^10^

Female BC mortality rates differ significantly between Caucasian women and African American (AA) women.^11^ Despite a lower overall incidence of BC, the mortality rate of AA women from BC is 42% higher compared to white women.^12^ In particular, a difference in the incidence and BC mortality exists between AA and Caucasian women below the age of 40 years old and then gradually diminishes during the later years.^11^, ^12^ AA women have higher rates of BC‐related mortality compared to Caucasian women, in part, because they have a higher incidence of triple negative BC (TNBC).^13^ TNBC represents roughly 10% of all BC diagnoses, yet TNBC makes up 21% of the BC cases seen in AA women.^14^ TNBC is an aggressive subtype that shows no expression of ER or PR, and lacks HER2 overexpression or gene amplification.^15^ Based on the gene expression profile, TNBC is further classified into basal‐like 1 (BL1), and 2 (BL2), mesenchymal (M), mesenchymal stem‐like (MSL), immunomodulatory (IM), and luminal androgen receptor (LAR) subtypes. DNA damage response pathways, ATR and BRCA, are greatly enhanced in the BL1 subtype gene ontologies. In BL2, myoepithelial markers such as TP63 and MME are expressed as well as distinct gene ontologies associated with growth factor signaling pathways. Both M and MSL subtypes display gene ontologies enriched in motility, extracellular matrix (ECM) remodeling, cell differentiation pathways, and epithelial to mesenchymal transitions (EMTs). In contrast to the M subtype, the MSL subtype displays decreased levels of proliferation genes and claudin protein, which is linked with genes associated with mesenchymal stem cells. In the IM subtype, gene ontologies related to immune signaling pathways are elevated. Gene ontologies associated with the LAR subtype demonstrate robust steroid synthesis and androgen/estrogen metabolism. As compared to other TNBC subtypes, the LAR subtype also expresses greater levels of androgen receptors.^16^, ^17^ The treatment response rate for the BL1, BL2, LAR, and MSL subtypes, are 52%, 0%, 10%, and 23% respectively.^18^ Figure 1B shows the genetic ontologies of TNBC genetic subtypes. Moreover, intra‐ and inter‐tumoral phenotypic and genotypic heterogeneity, and plasticity is often present in TNBC,^19^, ^20^, ^21^, ^22^, ^23^, ^24^, ^25^ which leads to drug resistance and tumor relapse. Although the prevalence of TNBC is only ~15%, the prognosis is usually poor due to metastases in patients diagnosed with TNBC.^13^, ^26^ TNBC's poorer outcomes are attributed to heterogeneity and some unique characteristics, as displayed in Figure 1B. Furthermore, the median overall survival (OS) in metastatic TNBC (mTNBC) is approximately 18 months, whereas in luminal BC, it is >5 years.^27^ Following standard of care neoadjuvant chemotherapy (NACT), TNBC patients demonstrate higher rates of relapse and distant metastases.^28^, ^29^ It is observed that pre‐menopausal, young AA women are disproportionately affected by mTNBC.^30^ This discrepancy can be attributed to a myriad of factors.

### 1.1 Cancer disparity

Cancer disparity is a significant issue affecting ethnic minorities, as well as socially and fiscally disadvantaged groups of people diagnosed with cancer.^31^ Despite the availability of a variety of cancer treatments, cancer disparities among ethnic minorities remains problematic and a major public health challenge.^32^ Compared to women of Caucasian descent, ethnic minorities and other medically underserved populations experience a disproportionate cancer burden for several cancer types.^31^ This is reflected by data indicating that AA women have the highest cancer mortality rate, compared to other ethnicities.^32^, ^33^, ^34^ AA and Hispanic cancer patients experience significant financial impact twice as often as Caucasian cancer patients, which causes issues with medication adherence.^34^ Societal barriers and inequities in accessing quality health care contribute to a disproportionate number of cancer morbidity and mortality among ethnic minorities.^32^, ^34^ Cancer health disparities not only negatively impact OS but also contribute to increased economic burden on the healthcare system. Therapies or other interventions that could decrease the ethnic disparity in TNBC, could have enormous fiscal implications by decreasing annual medical expenditures among such cancer patients.^31^, ^34^

### 1.2 BC disparity

BC is the leading type of cancer among AA women, with an estimated 36 260 new cases expected to be diagnosed in 2022.^31^, ^33^ In 2019, BC mortality rates have exceeded lung cancer mortality rates in AA women.^33^, ^34^ Furthermore, AA women less than 40 years of age (premenopausal women) have higher rates of morbidity, compared to premenopausal Caucasian women.^34^ In particular, there are key differences in the distribution BC subtypes between AA and Caucasian women. For example, AA women are twice as likely as women of other ethnicities to be diagnosed with TNBC^35^ and 41% more likely to be diagnosed with inflammatory BC, which are considered aggressive subtypes of BC.^33^ Currently, the explanation for this discrepancy remains to be determined. Unfortunately, the disparity in incidence is reflected in BC mortality rates. Despite a slightly lower overall BC incident rate, AA women are 40% more likely to die from BC compared to Caucasian women.^32^ A number of studies indicate that the higher rate of BC mortality is due to multiple factors, such as an advanced stage cancer at diagnosis, higher prevalence of aggressive subtypes of the disease (TNBC), and limited access to new treatment interventions, according to the calendar‐period effect.^32^, ^34^ AA women are more likely to suffer from socioeconomic disadvantages, but genetic factors also increase their risk for biologically aggressive tumor subtypes.^31^, ^36^

The incidence of obesity is greater in AA women (49.6%) compared their Caucasian (42.2%) counterparts.^34^, ^36^ Clinical studies have reported that obese patients have higher plasma levels of certain proinflammatory cytokines, such as resistin, IL‐6 and CXCL8.^37^, ^38^, ^39^ These cytokines can activate various oncogenic pathways, thereby leading to the probability of poor clinical outcomes in AA women.^34^

There are cellular and molecular factors that significantly contribute to the disproportionate incidence of BC in AA women.^40^ Understanding the cellular and molecular disparity will be critical for the development of efficacious precision oncology to treat patients who are affected as a result of ancestral origin.^34^

## 2 TUMOR MICROENVIRONMENT

The tumor microenvironment (TME) has been postulated to play a vital role in BC disparity.^34^, ^35^, ^36^, ^40^, ^41^, ^42^ The TME consists of interactions between cellular and non‐cellular components that affect tumor progression. The cellular components are comprised of BC cells and stromal cells.^43^ The stromal cells in BC include M1 and M2 macrophages, N1 and N2 neutrophils, cancer‐associated fibroblasts (CAFs), CD8^+^ T lymphocytes, regulatory T lymphocytes (T_regs_), B lymphocytes, plasma cells, mature and immature dendritic cells, myeloid‐derived suppressor cells (MDSCs), mesenchymal stem cells, endothelial and lymph endothelial cells, and adipocytes.^44^ Acellular components include stromal proteins such as collagen I, laminin, fibronectin, and hyaluronan.^44^, ^45^ The most abundant stromal cell type in the TME are CAFs.^46^ These cells are implicated in a number of versatile processes within the TME that facilitate BC tumor progression and invasiveness. CAFs may communicate with cancer and immune cells through direct or indirect routes to remodel the ECM, induce angiogenesis, and facilitate infiltration pro‐tumorigenic leukocytes in the TME which ultimately creates a favorable tumor environment.^47^, ^48^, ^49^ This communication is achieved by direct cell to cell contact or indirectly through the secretion of several growth factors and cytokines such as IL‐1β, IL‐6, IL‐25, CXCL12, and CXCL16.^48^ At the creation of this review, definitive associations between ethnicity and CAFs within humans have yet to be explored and reported. Several of the cytokines linked to CAFs are observed at higher levels in AA women with TNBC.

Specific cytokines and chemokines within the TME modulate primary tumor growth and the formation of polyclonal metastases.^45^ The differential infiltration of immune cells in AA patients, compared to Caucasian patients, alters the tumor immune microenvironment (TIME), which can significantly affect the pathogenesis of BC.^41^, ^42^ Consequently, a thorough understanding of TIME is necessary to delineate its role in BC disparity in order to develop precise treatment.

### 2.1 Disparate TIME

#### 2.1.1 Cytokines and CC chemokines that shape the TIME

**FIGURE 2:**
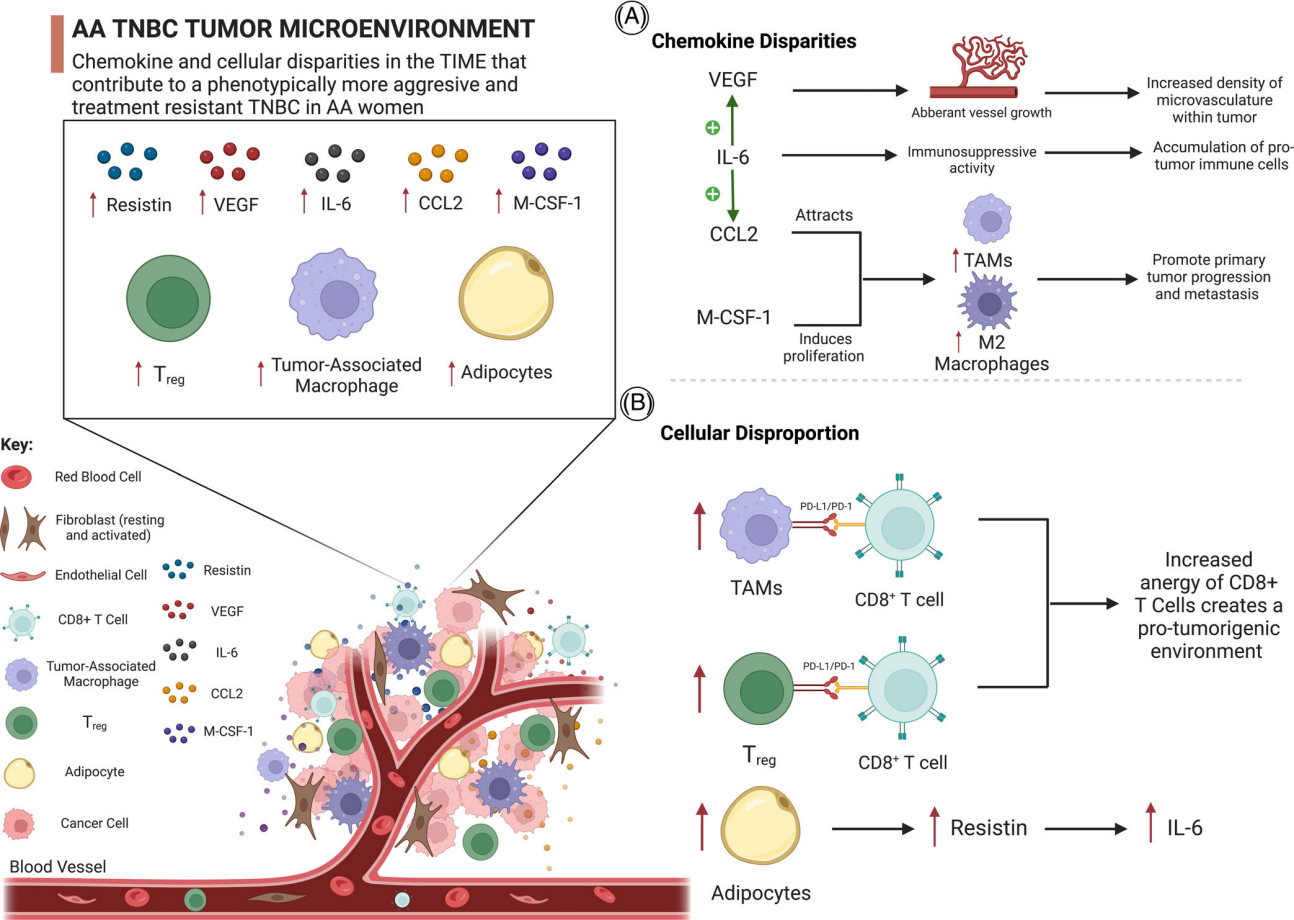
African American (AA) tumor microenvironment disparities associated with triple negative breast cancer (TNBC) tumors. (A) Shows the potential outcomes of certain chemokine levels in AA women's tumor microenvironment (TME) in terms of their potential outcomes. Elevated levels of these markers are associated with aggressive tumor phenotypes and a less favorable prognosis. (B) This illustration depicts the conceivable consequences associated with dysregulated cellular mechanisms in the TME of AA women. It is thought that these cells modify the antitumor immune microenvironment in the tumor, thus creating a pro‐tumorigenic environment and allowing cancer cells to survive in it

Cytokines are inflammatory mediators secreted by either BC or stromal cells that act in an autocrine or paracrine manner to augment tumor proliferation and metastasis.^50^ AA BC patients have increased plasma and TME levels of the cytokine, resistin, compared to Caucasian patients.^41^, ^51^ Resistin is derived from adipocytes and its plasma levels are higher in obese patients compared to their non‐obese counterparts.^52^ Obesity is a predisposing factor for the development of cancer in AA women.^36^, ^53^, ^54^ There is a significant positive correlation between plasma resistin levels and the size and stage of cancerous breast tumors and rate of lymph node metastasis.^55^, ^56^ In contrast, a lower plasma level of resistin correlated with a better OS and disease‐free survival compared to BC patients with high levels of resistin.^57^ Moreover, AA patients have higher plasma levels of the pro‐inflammatory cytokine, IL‐6.^58^, ^59^, ^60^ Cancer‐associated adipocytes and fibroblasts secrete inflammatory cytokines, including interleukin‐6 (IL‐6) and chemokine monocyte chemoattractant protein‐1 (MCP‐1) or CCL2.^61^, ^62^ IL‐6 is a major immunosuppressive cytokine within the TME that leads to immunoediting of the TIME,^54^ which can decrease the activity of anti‐tumor immune cells (dendritic cells, cytotoxic T‐cells)^63^, ^64^, ^65^ and promote the accumulation of pro‐tumor immune cells (tumor‐associated macrophages [TAM], myeloid‐derived suppressor cells [MDSCs], CD4^+^ T‐cells, and fibroblasts).^66^ Higher plasma levels of IL‐6 and resistin occurs more frequently in AA women compared to Caucasian women.^58^ A feed‐forward loop exists between IL‐6 and resistin, as they positively increase the levels of each other.^58^ Interestingly, IL‐6 induces the expression of vascular endothelial growth factor (VEGF) by activating the JAK‐STAT3 pathway.^58^, ^67^ Consequently, VEGF levels are also greater in AA patients compared to Caucasian patients, as indicated by the higher microvascular density in tumors of AA BC patients.^42^, ^67^ IL‐6 also induces the expression of the chemokine, CCL2.^68^, ^69^ Elevated levels of CCL2 provokes the accumulation of M2 macrophages in the TIME because they express the receptor for CCL2, also known as CCR2.^69^ An increase in the number of M2 or TAMs facilitates primary tumor progression and metastases.^70^ The expression of other CC chemokines, such as CCL17 and CCL25, in the TIME is negatively correlated with decreased OS in AA women. However, the levels of CCL8 are correlated with decreased OS in Caucasian women.^71^ Figure 2A displays the disparity of certain chemokines found within the TME of AA women and the associated outcomes of these chemokine imbalances.

#### 2.1.2 Disparity in TAMs

Pro‐tumor, polarized M2 macrophages demonstrate support of primary tumor growth and constitute the majority of the tumor‐resident immune cells present in solid malignancies. The reciprocal, paracrine interaction between TAMs and TNBC cells modulate the expansion of both cell types, which increases tumorigenesis and produces resistance to various types of treatment.^72^ In AA BC patients, there is a greater number of TAMs in TNBC tumors, compared to Caucasian patients.^42^, ^73^ TAMs have been reported to play an established role in facilitating tumor invasion, angiogenesis, immunosuppression, and metastasis.^70^ Soluble mediators secreted by TAMs play a role in the evasion of immune check points by cancer cells.^64^ Importantly, the plasma membrane of TAMs expresses the protein, programmed death ligand‐1 (PD‐L1). Consequently, a higher number of TAMs correlates with a higher expression of PD‐L1 in the TIME of TNBC tumors.^74^ This subsequently blunts the anti‐tumor response from cytotoxic CD8^+^ T lymphocytes by engaging with their PD‐1 receptor. Activation of the PD‐L1/PD‐1 axis under normal circumstances serves as a regulatory measure for peripheral T‐cell tolerance. Dysregulation of the expression of these receptors may cause excessive energy of CD8^+^ T lymphocytes. CD8^+^ T‐cells become unable to induce apoptosis or release lytic granules onto the cell of interest.^75^, ^76^ Additionally, T_regs_ express PD‐L1 which can induce energy of CD8^+^ T lymphocytes. The TME of AA BC patients have higher concentrations of chemokines, such as CCL2, resistin, and macrophage colony stimulating factor‐1 (M‐CSF‐1).^41^, ^42^, ^51^, ^77^ The increase in M‐CSF‐1 levels induces the migration of macrophages to the tumor site and the conversion of infiltrating monocytes from the circulation into macrophages within the TME.^41^, ^42^ Infiltrating monocytes and tumor‐resident macrophages also rapidly replicate due to the activation of their CSF‐1 receptor by M‐CSF‐1 in the TME, thus significantly increasing their population compared to other immune cells.^42^ Furthermore, this concurrently facilitates the decrease in the anti‐tumor response of CD8^+^ T lymphocytes by augmenting their inhibitory signaling through PD‐1 receptors, producing an exhaustion and energy of T lymphocytes.^76^ In contrast, blocking the CSF1/CSF1‐receptor axis potentiates the anti‐tumor cytotoxic activity of CD8^+^ T lymphocytes.^78^, ^79^ Overall, an increased frequency of TAMs and a higher microvasculature density (due to an increased level of VEGF in the TME of AA patients) contributes to a poorer prognosis.^42^, ^73^ In Figure 2B, AA women's TME is shown to exhibit cellular imbalances.

#### 2.1.3 CXC chemokines that regulate the TIME

CXC chemokines play a pivotal role in angiogenesis, cancer cell migration, invasion and metastasis.^50^ Notably, stromal cell‐derived factor‐1α (SDF‐1α), also known as chemokine CXCL12, is involved in the metastasis of many solid and hematological malignancies. CXCL12 is primarily secreted by CAFs into the TIME, where it activates the CXCR4 receptor.^80^ This receptor is present in cancer cells and is activated in a paracrine manner.^81^ Subsequently, the activated cancer cells migrate and invade locally into the vascular system as part of the metastatic cascade.^80^, ^81^ Alternatively, CXCL12 may interact with perlecan, a heparan sulfate proteoglycan within the basement membrane that functions structurally and sends signals, in the TME, which recruits CXCR4 receptor—containing regulatory T_regs_ that are Foxp3^+^ into the TME.^82^ T_regs_ are present in a significantly higher proportion of AA women compared to Caucasian women.^42^, ^79^ These cells have pro‐tumorigenic properties and can recruit MDSCs.^42^

## 3 THERAPEUTIC TARGETS

TNBC lacks the conventional, druggable targets found in other types of BCs which contributes to its therapeutic challenges and overall poor prognosis.^83^ The current mainstay of treatment typically involves a combination of NACT and surgery depending on the staging at diagnosis.^53^ The risk of BC mortality is significantly higher among AA women with TNBC compared to Caucasian women which is partially explained by the disparities in surgery and chemotherapy.^84^ As the dynamic role of the TME becomes better understood, therapeutic targets may be identified within this niche. It is possible that the disparities uncovered within the TME of AA women may provide a solution to address the adverse outcomes attributed to ethnicity in TNBC patients. This review will focus on the clinical trials investigating targeted chemokine/cytokine inhibition, immune checkpoint inhibition, and adoptive‐cell transfer therapy that address the TME discrepancies described previously. Nanotherapeutic and natural compound application within the TME of BC provides another approach for targeted treatment. Raju et al. and Dias et al. provide extensive reviews regarding nanotherapeutics and natural compound utilization in the TME, respectively.^85^, ^86^ The clinical trials discussed throughout this paper is summarized in Table 1.

**TABLE 1:** Summarizes the ongoing and completed clinical trials on potential therapeutic targets in the TME of TNBC patients presented throughout this paper

Type of therapeutic target	Identifier	Title	Phase of trial	Status	Year of registration
*Chemokine, cytokine, and downstream targets*					
IL‐6 Inhibition	NCT03424005	A Study Evaluating the Efficacy and Safety of Multiple Immunotherapy‐Based Treatment Combinations in Patients with Metastatic or Inoperable Locally Advanced Triple‐Negative Bread Cancer (Morpheus‐TNBC)	Ib/II	Active, not recruiting	2018
	NCT04333706	A Dose Finding Phase 1 of Sarilumab Plus Capecitabine in HER2/Neu‐Negative Metastatic Breast Cancer and a Single‐Arm, Historically‐Controlled Phase 2 Study of Sarilumab Plus Capecitabine in Stage I‐III Triple Negative Breast Cancer with High‐Risk Residual Disease (EMPOWER)	I	Active, recruiting	2020
JAK Inhibition	NCT03012230	Pembrolizumab and Ruxolitinib Phosphate in Treating Patients with Metastatic Stage IV Triple Negative Breast Cancer	I	Active, recruiting	2017
	NCT02928978	Ruxolitinib for Premalignant Breast Disease (TBCRC042)	1	Active, recruiting	2016
	NCT01562873	Ruxolitinib in Patients with Breast Cancer	II	Terminated	2012
	NCT02876302	Study of Ruxolitinib (INCB018424) with Preoperative Chemotherapy for Triple Negative Inflammatory Breast Cancer	II	Active, not recruiting	2016
STAT Inhibition	NCT03195699	Oral STAT3 Inhibitor, TTI‐101, in Patients with Advanced Cancers	I	Active, not recruiting	2017
VEGF Inhibition	NCT00600340	2‐Arm Trial of Paclitaxel Plus Bevacizumab vs. Capecitabine Plus Bevacizumab (TURANDOT)	III	Completed	2008
	EudraCT Number: 2006‐006058‐83	Phase II Randomized Trial of Combination Therapy of Paclitaxel and Bevacizumab Versus Paclitaxel, Capecitabine and Bevacizumab as First‐Line Treatment for Locally Recurrent or Metastatic Breast Cancer Patients with HER2/Neu Negative Tumor (ATX‐study)	I	Active, not recruiting	2007
	NCT05192798	Albumin‐Bound Paclitaxel Combined with Antiangiogenic Agents in First‐Line Treatment of Relapsed or Metastatic TNBC	II	Active, recruiting	2022
	NCT04427293	Preoperative Lenvatinib Plus Pembrolizumab in Early‐Stage Triple‐Negative Breast Cancer (TNBC)	I	Active, recruiting	2020
*Immune checkpoint inhibitors*					
PD‐l/PD‐L1 mAb Inhibition	NCT03036488	Study of Pembrolizumab (MK‐3475) Plus Chemotherapy vs Placebo Plus Chemotherapy as Neoadjuvant Therapy and Pembrolizumab vs Placebo as Adjuvant Therapy in Participants with Triple Negative Breast Cancer (TNBC) (MK‐3475‐522/KEYNOTE‐522)	III	Active, not recruiting	2017
	NCT02425891	A Study of Atezolizumab in Combination with Nab‐Paclitaxel Compared with Placebo with Nab‐Paclitaxel for Participants with Previously Untreated Metastatic Triple‐Negative Breast Cancer (IMpassion130)	III	Completed	2015
	NCT03125902	A Study of Atezolizumab and Paclitaxel Versus Placebo and Paclitaxel in Participants with Previously Untreated Locally Advanced or Metastatic Triple Negative Breast Cancer (TNBC) (IMpassion131)	III	Active, not recruiting	2017
	NCT03197935	A Study to Investigate Atezolizumab and Chemotherapy Compared with Placebo and Chemotherapy in the Neoadjuvant Setting in Participants with Early Stage Triple Negative Breast Cancer (IMpassion031)	III	Completed	2015
	NCT02819518	Study of Pembrolizumab (MK‐3475) Plus Chemotherapy vs. Placebo Plus Chemotherapy for Previously Untreated Locally Recurrent Inoperable or Metastatic Triple Negative Breast Cancer (MK‐3475‐355/KEYNOTE‐355)	III	Active, not recruiting	2016
PD‐1/PD‐L1 mAB with ADC or PPARi inhibition	NCT04468061	Sacituzumab Govitecan +/‐ Pembrolizumab in Metastatic TNBC	II	Active, recruiting	2020
	NCT02574455	Trial of Sacituzumab Govitecan in Participants with Refractory/Relapsed Metastatic Triple‐Negative Breast Cancer (TNBC) (ASCENT)	III	Completed	2015
	NCT03310957	Safety and Efficacy of SGN‐LIVIA Plus Pembrolizumab for Patients with Locally‐Advanced or Metastatic Triple‐Negative Breast Cancer	Ib/II	Active, recruiting	2017
	NCT03167619	Phase II Multicenter Study of Durvalumab and Olaparib in Platinum Treated Advanced Triple Negative Breast Cancer (DORA)	II	Completed	2017
*Adoptive cell transfer bated therapy*					
Tumor‐infiltrating lymphocyte adoptive cell transfer	NCT01174121	Immunotherapy Using Tumor Infiltrating Lymphocytes for Patients with Metastatic Cancer	II	Active, recruiting	
	NCT03645928	Study of Autologous Tumor Infiltrating Lymphocytes in Patients with Solid Tumors	II	Active, recruiting	2010
	NCT03610490	Autologous Tumor Infiltrating Lymphocytes MDA‐TIL in Treating Patients with Recurrent or Refractory Ovarian Cancer, Colorectal Cancer, or Pancreatic Ductal Adenocarcinoma	II	Active, recruiting	2018
CAR‐T adoptive cell transfer	NCT01837602	cMet CAR RNA T Cells Targeting Breast Cancer	I	Completed	2013
	NCT05341492	EGFR/B7H3 CAR‐T on Lung Cancer and Triple Negative Breast Cancer	I	Active, recruiting	2022
	NCT05274451	A Study of Investigate LYL797 in Adults with Solid Tumors	I	Active, recruiting	2022

### 3.1 Chemokine and cytokine therapy

The TME of AA women with TNBC display significantly higher levels of IL‐6 and other pro‐inflammatory cytokines, like TNF and leptin, compared to Caucasian women. It is hypothesized that this is due to health disparities predisposing AA patients with TNBC to obesity and diabetes which supports a pro‐inflammatory environment.^87^ Increased IL‐6 in TNBC is negatively correlated to unfavorable outcomes.^51^, ^88^ IL‐6 is a cytokine that regulates a number of physiological processes, including immune and inflammatory responses.^68^, ^88^ The IL‐6 axis is frequently dysregulated in cancer, which promotes pro‐tumor effects such as cancer growth, migration, and metastasis by activating the IL‐6/JAK/STAT3 pathway.^89^, ^90^, ^91^ Although IL‐6/JAK/STAT3 inhibitors have yet to be approved by the FDA for BC, there are several ongoing clinical trials evaluating the efficacy of compounds that inhibit this pathway in TNBC^92^, ^93^, ^94^, ^95^, ^96^, ^97^, ^98^ which could be useful in treating AA women. Additionally, VEGF expression is greater in the TME of AA women compared to Caucasian women, and this could represent a potential therapeutic target for TNBC treatment.^73^ VEGF is a downstream growth factor, and its expression is induced by hypoxia, other growth factors, oncogenes, and IL‐6 which facilitates angiogenesis and lymphangiogenesis.^99^, ^100^ Increased expression of VEGF in solid tumors is positively correlated with more aggressive and metastatic disease.^101^ Targeting anti‐angiogenic pathways has remained a focus in clinical trials over the past decades as a potential treatment option for cancers that express high levels of VEGF.^102^

#### 3.1.1 IL‐6 Inhibition

Tocilizumab, an anti‐IL‐6Ra monoclonal antibody that binds and neutralizes IL‐6R,^103^ is currently being investigated in tandem with atezolizumab, a humanized IgG1 monoclonal anti‐PDL‐1 antibody,^104^ and nab‐paclitaxel, an albumin‐bound formulation of paclitaxel that inhibits the depolymerization of microtubules in the G2 and M phases of the cell cycle.^105^ This combination will be assessed in a phase Ib/II trial in patients with metastatic or inoperable locally advanced TNBC.^92^ Similarly, sarilumab, an anti‐IL‐6Ra mAb, in combination with capecitabine, is being evaluated in stage I‐III TNBC and metastatic TNBC patients.^93^ Studies have demonstrated that tocilizumab can attenuate the aggressive features in Castleman disease, a lymphoproliferative disorder,^106^, ^107^ due to blocking the IL‐6/IL‐6Ra signaling cascade.^108^ Both tocilizumab and sarilumab have been approved by the United States Food and Drug Administration (FDA) for the treatment of rheumatoid arthritis^108^ and their evaluation in TNBC patients is expected to end in 2023.^92^, ^93^

#### 3.1.2 JAK and STAT inhibitors

Downstream targets of the IL‐6 cascade, such as Janus tyrosine kinase 1 and 2 (JAK1/2) and Signal transducer and activator of transcription 3 (STAT3) are also being clinically evaluated for their anti‐tumor efficacy in BC.^94^, ^95^, ^97^, ^98^ The JAK inhibitors (abrocitinib, baricitinib, delgocitinib, fedratinib, filgotinib, oclacitinib, pacritinib, peficitinib, ruxolitinib, tofacitinib, and upadacitinib) have been approved by the FDA for the treatment of a variety of inflammatory diseases.^109^ Certain IL‐6 inhibitors are now being evaluated for the treatment of BC.^94^, ^95^, ^96^, ^98^ Used as monotherapy in a phase II clinical trial, ruxolitinib, an oral tyrosine kinase inhibitor of JAK1 and JAK2, was not efficacious for the treatment of metastatic TNBC.^96^ This prompted the investigation of ruxolitinib use in combination with other cancer therapeutics for the treatment of BC. One such phase II clinical trial is looking at the effects of ruxolitinib used in combination with paclitaxel, followed by the mainstay chemotherapeutic drugs for BC, doxorubicin and cyclophosphamide, for the treatment of TNBC.^98^ Another phase I clinical trial is assessing the use of ruxolitinib phosphate with pembrolizumab, an anti PD‐1 agent, for the treatment of metastatic stage IV TNBC.^94^ Additionally, a phase I clinical trial is evaluating the efficacy of a direct STAT3 inhibitor called compound TTI‐101 that is set to end in 2023.^97^ Decreasing the interaction of IL‐6 with its receptor and its downstream pathways may provide a particularly useful treatment modality for AA women with TNBC.

#### 3.1.3 VEGF inhibitors

Currently, IFN‐α and bevacizumab, a monoclonal VEGF antibody, are among first line drugs for the treatment of metastatic RCC.^110^ The results of phase III clinical trials have reported an increased median survival of patients with colorectal cancer, and lung cancer, as well as increased progression free survival of BC patients when bevacizumab was combined with chemotherapy. However, there were no significant differences in median survival when bevacizumab was used as monotherapy.^111^ Several ongoing clinical trials are evaluating the efficacy of anti‐VEGF drugs in combination with other anticancer drugs as potential treatment options for BC tumors overexpressing VEGF.^112^, ^113^, ^114^ Compared to chemotherapy alone, the addition of VEGF inhibitor, bevacizumab, to paclitaxel or capecitabine treatment regimens significantly increases progression free‐survival and the objective response rate in HER2 negative locally recurrent or metastatic BC patients.^111^ A randomized phase II clinical trial evaluated the efficacy of bevacizumab and paclitaxel, with or without capecitabine, for the treatment of HER‐2 negative local or recurrent metastatic BC. Patients treated with the combination of bevacizumab, paclitaxel, and capecitabine had significantly longer progression free‐survival times and response duration, compared to patients treated with only bevacizumab and paclitaxel.^112^ Another phase II clinical trial will evaluate the efficacy of apatinib, a VEGFR‐2 tyrosine kinase inhibitor, combined with albumin‐bound paclitaxel, as well as the combination of albumin‐bound paclitaxel and bevacizumab compared to albumin‐bound paclitaxel monotherapy.^113^ Finally, an ongoing phase I trial will assess the efficacy of levatinib, an inhibitor of VEGF and multiple other tyrosine kinase receptors^115^ and pembrolizumab, a PD‐1 receptor inhibitor prior to surgery for untreated TNBC.^114^

### 3.2 Immune checkpoint inhibitors

Elevated levels of certain chemokines and cytokines in the TME of AA BC patients suggests that specific immunotherapies may be a potential treatment option. Immune cells, transcription factors, and certain cytokines activate immune checkpoints, such as PD‐L1, in response to inflammation in the TME. Tumors overexpress PD‐L1 to evade the death of cancer cells by CD8^+^ T lymphocytes.^116^ Normally, cytotoxic T‐cells carry out a process known as “target‐cell death” by recognizing malignant cells, creating pores in the cell membrane, and releasing granules that contain lysis‐inducing enzymes. T‐cells express immune checkpoint proteins like PD‐1 and CTLA‐4 which help regulate the immune response by preventing the activation of T‐cells and targeted cell‐death of normal and healthy cells. If a checkpoint protein binds with its corresponding partner protein, an “off” signal is relayed to the T‐cell preventing its activation.^117^ Although vital for the maintenance of normal tissues, certain cancers, such as melanoma, lung cancer, and subsets of TNBC overexpress PD‐L1 to escape T‐lymphocyte‐mediated destruction.^116^ By targeting and inhibiting these immune checkpoints, T‐cells can be rescued from exhaustion and energy, thus potentiating their anti‐cancer effects.^117^ Certain subsets of TNBC have been shown to have a high tumor mutation burden and high tumor‐infiltrating lymphocytes, which are also present in melanoma and lung cancer.^118^ Patients with melanoma and lung cancer have benefited from treatment with immune checkpoint inhibitors, and it is possible that these drugs could be potential treatments for TNBC.

#### 3.2.1 PD‐1 Blockers

Immunotherapy targeting the PD‐1/PD‐L1 interaction between cells may be a treatment for patients with TNBC. In fact, clinical trials have been conducted to evaluate the efficacy of PD‐1/PD‐L1 inhibitors for the treatment of TNBC. The PD‐1/PD‐L1 inhibitors, atezolizumab and pembrolizumab, were evaluated in phase III clinical trials for the treatment of metastases in TNBC patients (NCT03036488 and NCT02425891).^119^, ^120^ The efficacy of atezolizumab and nab‐paclitaxel was evaluated in patients with untreated, locally advanced or metastatic TNBC, in a clinical trial called Impassion130. Compared to chemotherapy alone, the combination of PD‐L1 blockade with nab‐paclitaxel produced a significant improvement in progression—free survival, especially in PD‐L1 positive patients.^119^ Atezolizumab was granted accelerated approval by the US FDA for the treatment of metastatic TNBC but was later withdrawn after a subsequent study (Impassion131) failed to show significant improvement in progression—free survival and OS after treatment with atezolizumab.^121^ The administration of atezolizumab and nab‐paclitaxel, in the setting of neoadjuvant early‐stage TNBC, significantly improved define pCR (pCR), compared to placebo in the clinical trial, Impassion031.^122^

Pembrolizumab is a monoclonal antibody that specifically binds to PD‐1, preventing its interaction with its ligand, PD‐L1.^123^ In a recent clinical trial, pembrolizumab, in combination with chemotherapy (nanoparticle albumin‐bound paclitaxel, paclitaxel, or gemcitabine–carboplatin), produces a significantly longer progression—free survival in a subset of patients with TNBC, compared to patients treated with chemotherapy alone. The expression of PD‐L1 and a combined positive score (CPS) of at least 10 were the requirements for the cohort. The CPS was equal to the number of PD‐L1‐staining tumor cells, lymphocytes, and macrophages, divided by the total number of viable tumors cells, multiplied by 100.^120^ This treatment combination was approved by the FDA in 2020 for patients meeting these requirements.^124^ The response rates of these treatments were significantly dependent on the level of PD‐L1 expression and the number of tumor‐infiltrating lymphocytes (TILs) present in the TME of the patients.^125^ The administration of the PD‐1/PD‐L1 inhibitors (pembrolizumab and atezolizumab) as monotherapy demonstrate encouraging results, however, the most efficacy is observed in patients that were given an additional chemotherapeutic drug.^126^ These results led to a significant increase in the clinical evaluation of immune checkpoint inhibitors, in combination with other anti‐cancer drugs, such as PARP inhibitors, antibody‐drug conjugates, and chemotherapy such as alkylating agents, antimetabolites, plant alkaloids, and anthracyclines.^118^ A phase II trial that will end in 2024, will determine the progression free survival of patients treated with sacituzumab govitecan and pembrolizumab, compared to sacituzumab govitecan alone, in PD‐L1 negative mTNBC.^127^ Sacituzumab govitecan is an antibody drug conjugate (ADC) designed to target human trophoblast cell‐surface antigen 2 (Trop‐2), with a hydrolysable active metabolite of irinotecan attached for release at the target. Sacituzumab govitecan monotherapy produced a greater progression—free survival and OS compared to chemotherapy, a treatment that has been approved by the FDA in mTNBC patients.^128^ Similarly, another ongoing trial is evaluating the efficacy of the ADC, ladiratuzumab vedotin, as a first‐line treatment for locally advanced or mTNBC in combination with pembrolizumab (NCT03310957).^129^ Ladiratuzumab vedotin targets the protein LIV‐1, a plasma membrane bound zinc‐transporter protein. Attached onto the antibody is a protease‐cleavable monomethyl auristatin E (MMAE), a drug that disrupts microtubules by inhibiting tubulin polymerization.^129^ The efficacy of the combination of olaparib, a PARP inhibitor, in combination with durvalumab, a PD‐1 inhibitor, was evaluated as maintenance therapy, compared to platinum—sensitive TNBC, in a phase II clinical trial that was completed in September 2022.^130^, ^131^ In preclinical studies, PARP inhibitors (pamiparib), combined with a PD‐L1 blocker, significantly inhibited tumor growth in mouse cell lines.^132^ The efficacy of other drugs targeting the PD‐1/PD‐L1 system are currently undergoing phase II and III clinical trials.^133^ Overall, it is possible that certain types of immunotherapies could potentially be of significant benefit to AA women who display higher levels of TILs in their TME. In combination with other drugs, immune checkpoint inhibitors may provide a promising treatment option for TNBC patients, particularly those that express high levels of PD‐L1, an increased number of TILs, or have a high tumor mutational burden.

### 3.3 Adoptive cell transfer‐based therapy: TILs and chimeric antigen receptor T cell therapy

The TIME of AA women contain a greater number of T_reg_ cells compared to Caucasian women.^42^ Under normal conditions, T_reg_ cells mediate tolerance and control autoimmunity by regulating the production of T‐cells, chemokines, and cytokines.^134^ Cancer cells can adapt to their environment to ensure their survival in the presence of anti‐tumor mechanisms present within the body.^135^ Some of these biochemical adaptions affect tumor infiltrating T_regs_ by promoting their expansion and differentiation, increasing the levels of immunosuppressive chemokines and increasing the probability of cancer cell survival and proliferation.^136^ The presence of a large number of T_regs_ in the TME increases immunosuppression, which decreases the efficacy of chemotherapy and radiotherapy.^137^

#### 3.3.1 Tumor‐infiltrating lymphocyte adoptive cell transfer

**FIGURE 3:**
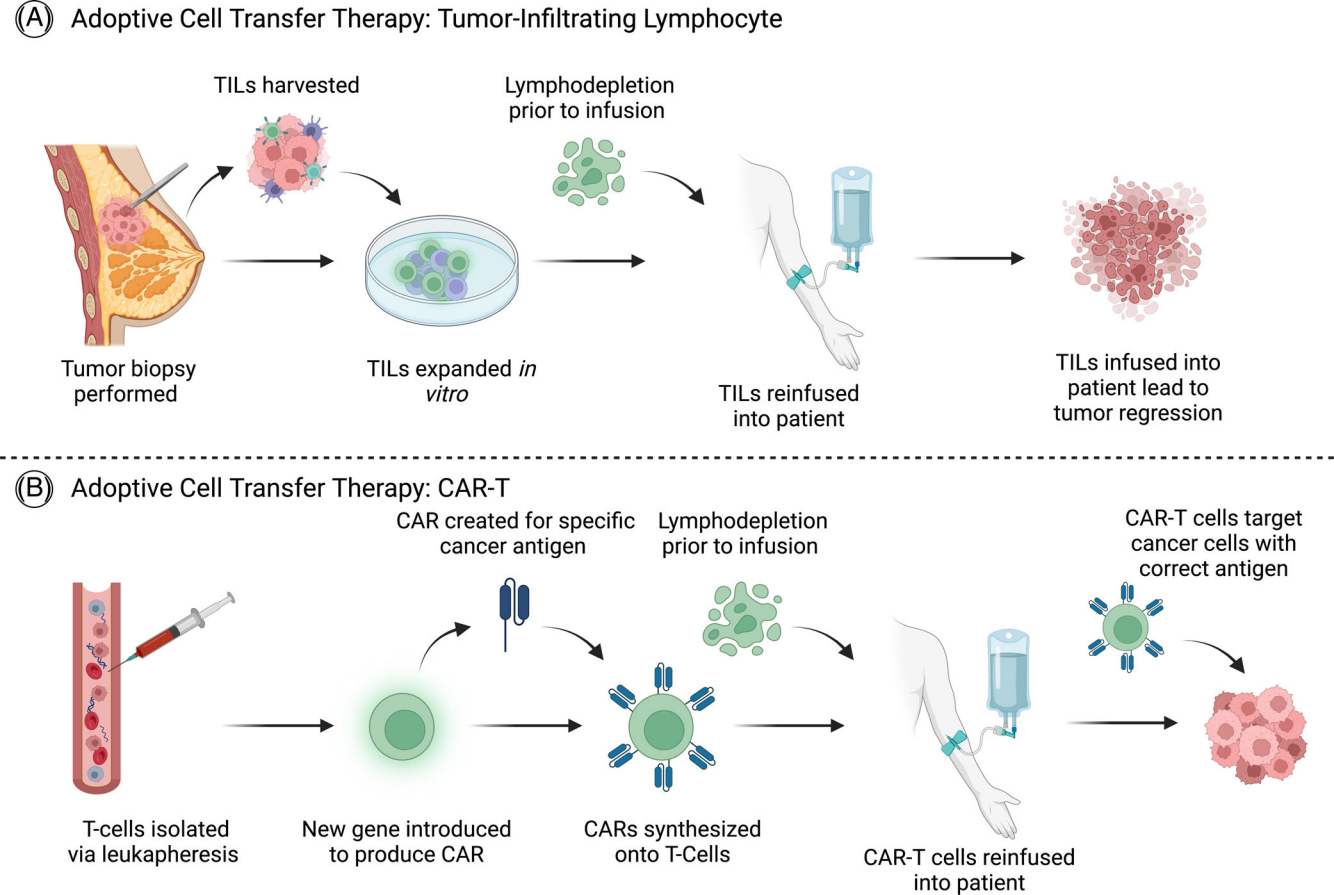
Adoptive cell transfer techniques currently undergoing clinical trials for triple negative breast cancer (TNBC). (A) Shows the step‐by‐step process that patients must undergo prior to undergoing TIL adoptive cell transfer procedure. (B) Shows how in order for chimeric antigen receptor T cell therapy (CAR‐T) adoptive cell transfer therapy to be performed, a series of steps must be taken in order for the chimeric antigen receptor to be created on an autologous T‐cell

Tumor‐infiltrating lymphocyte cell‐transfer therapy is another therapeutic option for the treatment of TNBC patients regardless of if the number of TILs is low or if their anticancer capability is diminished by a highly immunosuppressive TME. This paradigm involves harvesting a patient's TILs, expanding the population of neoantigen‐specific TILs, and reintroducing those TILs back into the patient to help decrease tumor growth.^138^ Several preclinical trials have reported promising results for adoptive cell transfer as an emerging anti‐cancer therapeutic,^139^ although clinical trials are still in the early stages.^138^, ^140^, ^141^ Currently, there is an ongoing phase II clinical trial that is assessing the efficacy of reintroduced TILs via adoptive cell transfer, in combination with pembrolizumab. The patients with TILs that have immunogenicity toward somatic mutations in cancer cells and demonstrate robust immunity underwent adoptive transfer. Of the six patients who underwent treatment for metastatic BC, three had tumor regression and one patient had a complete response.^138^ Other clinical trials are currently recruiting patients to evaluate the efficacy of TIL adoptive cell transfer in TNBC patients. A phase II study that will be completed in 2023 is currently recruiting patients to evaluate the safety and efficacy of TIL therapy in mTNBC patients. Tumor lymphocytes will be harvested from tumor tissue and the infused autologous LN‐145 product (amplified patient tumor TILs with stimulating cytokines) will occur on the first hospital day.^140^ Another phase II trial that will be terminated in 2024, is currently recruiting patients with refractory or relapsed cancer, including TNBC. The LN‐145 or LN‐145‐S1 product will be infused back into patients after initial tumor resection.^141^ Both trials will evaluate the validity of TIL therapy in patients with mTNBC which may provide another treatment paradigm to manage TNBC. Figure 3A represents a schematic of the process patients encounter when undergoing tumor‐infiltrating lymphocyte adoptive cell transfer.

#### 3.3.2 Chimeric antigen receptor T cell therapy adoptive cell transfer

Chimeric antigen receptor T cell therapy (CAR‐T) therapy is another adoptive cell transfer‐based therapy that has shown great success and has been approved for the treatment of several hematological malignancies.^142^ Similar to TIL adoptive cell transfer therapy, the T‐cells of the patient are harvested, further manipulated ex vivo to recognize tumor‐associated antigens via synthetic receptors, then amplified ex vivo and re‐introduced into the patient.^143^ Because of the success in treating certain hematologic malignancies, researchers have detected antigens exclusive to solid tumors such as TNBC.^144^ A phase I trial conducted at the University of Pennsylvania to evaluate the safety and efficacy of CAR‐T cell therapy against C‐met, a cell surface molecule present in several solid malignancies including BC.^145^, ^146^ After this study was concluded, inflammatory responses within the tumor were noted.^146^ A trial that will end in 2025 is currently recruiting patients with EGFR/B7H3 positive TNBC to evaluate the safety and objective response rate of CAR‐T cell therapy.^147^ Similarly, another trial will evaluate the safety and tolerability of CAR‐T cell therapy in receptor tyrosine kinase like orphan receptor 1 (ROR1) positive refractory or relapsed TNBC. This trial will be divided into two parts: Part 1 will determine dose tolerability through a dose escalation phase and Part 2 will determine the safety, efficacy and duration of response in TNBC and non‐small cell lung cancer (NSCLC) patients.^148^ Although preclinical and clinical trials have reported encouraging results in the treatment of solid tumors using CAR‐T therapy, there are still several challenges associated with this paradigm such as: poor infiltration of CAR‐T cells into solid malignancies, the presence of immunosuppressive TMEs, and off‐target side effects.^149^ It is possible that reintroducing anti‐tumorigenic T‐cells could provide a therapeutic option for AA women with immunosuppressed TMEs surrounding their TNBC cells. As shown in Figure 3B, patients will undergo CAR‐T therapy and their reinfused T‐cells will be engineered to have a synthetic CAR.

## 4 LIMITATIONS

Evidence suggests AA women with nonmetastatic TNBC were less likely to receive surgery or chemotherapy compared to Caucasian women. Additionally, they were more likely to die from the disease than their Caucasian counterparts. Grigs et al. also found that adjuvant chemotherapy dose proportions and intensities were lower in AA women when compared with Caucasian women.^150^ The AA women may have differences in cancer outcomes from Caucasian women due to inadequate surgical resection/adjuvant treatment, a delay in adjuvant chemotherapy, or suboptimal cancer treatment administration.^151^, ^152^ Although this review focuses on the TME, further research into tumor biology, metastasis versus non‐metastasis, treatment effectiveness, and access to care in AA women with TNBC may provide insight into why these women had significantly poorer therapeutic outcomes than Caucasian women with the same cancer.

Cancer therapies that target the TME also present challenges. When cancer cells die from chemotherapy or radiotherapy, they release the high‐mobility group box 1 (HMGB1) protein. HMGB1 acts as a danger signal, activating Toll‐like receptor‐4 (TLR4), which activates a protective immune response, prolonging antitumor protection, thus decreasing the effectiveness of chemotherapy and radiotherapy.^153^ In the same way, TILs are personalized immunotherapies that are tailored to the patient's malignancy. Unlike adjuvant chemotherapy or monoclonal antibodies, TILs products are made to order, which means they are relatively expensive when compared to mass‐produced adjuvant chemotherapy. Additionally, the longer production time of TILs products may also be disadvantageous for AA patients with aggressive cancer.^154^ The selection of an appropriate target is a major challenge for the success of CAR‐T cell adoptive therapy. There are multiple factors that impact the choice of a target, including antigen expression levels, heterogeneity of tumor antigens, and efficient antigen presentation.^155^, ^156^ Similarly, CAR‐T therapy is impeded by immune‐suppressive TME, which inhibits infiltration of CAR‐T cells. For adoptive CAR‐T to be successful, the TME may need to be remodeled to promote anticancer immunity and boost the success rate of treatments.^155^

## 5 CONCLUSIONS

AA women experience phenotypically more aggressive TNBC with an associated poorer prognosis. More research support is essential for the prevention and treatment of TNBC and should be of high priority. This is vital to combat racial disparities in clinical outcomes and reduce mortality. As overall BC incidence remains considerably lower among AA women compared to Caucasian women, providing AA women with timely, high quality medical care can efficiently impact the racial disparity by reducing TNBC mortality. Understanding the cellular and molecular disparities within the TME of AA women will provide an essential key in addressing the unfavorable outcomes seen in TNBC. The atypical TME in AA women deliver a unique opportunity to implement precision oncology to decrease morbidity and provide treatment for those harboring chemokine and cellular imbalances. Cho et al. suggested that improving adherence and efficacy of TNBC treatments is crucial to reducing disparities.^84^ A multitude of promising clinical trials are currently underway exploring the safety and efficacy of new therapeutics and old therapeutics as monotherapy or in combination. This is a multi‐factorial issue, however, understanding of the TME and further advancement of targeted personalized medicine may provide additional benefit to those affected by TNBC.

## AUTHOR CONTRIBUTIONS

**Kelsee Keyshu Zajac:** Software (lead); writing – original draft (Equal); writing – review and editing (lead). **Saloni Malla:** Writing – original draft (supporting). **Ramapuram Jayachandra Babu:** Writing – original draft (supporting); writing – review and editing (supporting). **Dayanidhi Raman:** Conceptualization (equal); funding acquisition (equal); writing – original draft (Equal). **Amit K. Tiwari:** Conceptualization (equal); funding acquisition (equal); project administration (lead); writing – review and editing (supporting).

## CONFLICT OF INTEREST

The authors have stated explicitly that there are no conflicts of interest in connection with this article.

## ETHICS STATEMENT

The authors are accountable for all aspects of the work in ensuring that questions related to the accuracy or integrity of any part of the work are appropriately investigated and resolved.

## Data Availability

Data sharing is not applicable to this article as no new data were created or analyzed in this study.
